# Accumulation and within-mushroom distribution of elements in red cracking bolete (*Xerocomellus chrysenteron*) collected over the extended period from compositionally contrasting substrates

**DOI:** 10.1007/s10661-023-11786-6

**Published:** 2023-09-06

**Authors:** Alexandre V. Andronikov, Irina E. Andronikova, Ondrej Sebek, Eva Martinkova, Marketa Stepanova

**Affiliations:** Division of Geochemistry and Laboratories, Czech Geological Survey, Geologicka 6, 15200 Prague, Czech Republic

**Keywords:** Trace elements, Uptake, Translocation, Soil, Mushroom, Bedrock

## Abstract

We conducted a study of elemental compositions of *Xerocomellus chrysenteron* samples accompanied by samples of related substrate soils. All samples were collected during the harvesting seasons 2021 and 2022 from three forested sites almost unpolluted by recent human activities and underlain by contrasting bedrock (granite, amphibolite, and serpentinite). Elements such as Ag, Cd, K, P, Rb, S, Se, and Zn were the main elements enriched in the mushroom’s fruiting bodies relative to the substrate. Concentrations of most elements in mushrooms were not site-dependent, with only Ag, As, Rb, and Se concentrations significantly depending on the bedrock composition. Some elements analyzed in mushrooms displayed temporal features, but such features were not systematic and varied for each element. Most analyzed elements were distributed unevenly within the mushroom’s fruiting bodies, with apical parts generally enriched in mobile elements. Mushrooms influenced concentrations of Ag, Cd, K, and Rb and a few other elements in the substrate via uptake, but such influence was very limited and can be responsible for only 2.5–11.5% of total depletion of the affected substrate in the named elements.

**Supplementary Information:**

The online version contains supplementary material available at 10.1007/s10661-023-11786-6.

## Introduction

Ectomycorrhizal basidiomycetes effectively transport elements from the lithosphere into the biosphere and form an ecological group of soil fungi participating significantly in mineral weathering (Gadd, 2007). Mushrooms readily accumulate various elements (including toxic) from the substrate doing this much more effectively than vascular plants and playing therefore an important role in biochemical cycling (e.g., Falandysz & Borovička, 2013; Kalač, 2009, 2010; Zocher et al., 2018). Concentrations of trace metals in mushroom fruiting bodies can serve as a signature for environmental pollution (e.g., Brzezicha-Cirocka et al., 2016; Ediriweera et al., 2022; Ingrao et al., 1992; Świsłowski & Rajfur, 2018). Mushrooms can grow in different environments, i.e., in both relatively unpolluted (forests, parks) and significantly polluted (industrial wastes) areas. Since mushrooms are getting their nutrients mostly from the substrate and not from the atmosphere (Arnott, 1995; Dusengemungu et al., 2021;Gadd, 1999, 2007; Gadd et al., 2014; Landweert et al., 2001), soil type and a degree of its pollution can significantly affect chemical composition of mushrooms (Chojnacka et al., 2012, 2013; Damodaran et al., 2014; Jarzyńska et al., 2012; Kojta et al., 2016; Zhang et al., 2010; Zsigmond et al., 2020). An initial step in getting insight into the elemental composition of mushrooms and their bioaccumulative (or bioconcentration, i.e., soil/organism interaction) potential is the examination of fruiting bodies assisted with the study of the underlain substrate.

Since mushrooms as a group and Boletaceae in particular may serve as a monitoring tool of environmental pollution level (Chojnacka et al., 2012, 2013; Jarzyńska et al., 2012), knowledge about elements’ bioconcentrations by mushrooms from soil as well as about within-mushroom distribution of the elements are important for environmental studies. Extension of such examinations to a longer period and over areas underlain by different bedrock may offer a new perspective for understanding the partitioning of elements between biologic and mineral geochemical reservoirs in the Critical Zone. As of now, only a few works are dealing with the long-term observations on the elemental compositions of Boletaceae mushrooms and, in particular, *Boletus edulis* and *Xerocomus subtomentosus* (Chojnacka et al., 2013; Collin-Hansen et al., 2005; Zhang et al., 2010). Those authors showed that environmental conditions (soil composition, a degree of the pollution, weather, and biological factors) can cause some variation in elemental compositions of the mushrooms fruiting bodies.

In order to stay in line with and to extend the long-term monitoring studies of Boletaceae mushrooms, we conducted a study of samples of *Xerocomellus chrysenteron* collected over the extended period (two mushroom harvesting seasons) from three sites underlain by contrasting bedrock. *Xerocomellus chrysenteron* represents one of the most widespread and most widely consumed Boletaceae mushrooms in the Czech Republic (Antonín et al., 2019; Knauerová et al., 2020). It grows from late June to early October mostly in coniferous forests. The aims of the present study were as follows: (i) to reveal bioaccumulative potential of *X. chrysenteron* growing on contrasting substrates in the forested unpolluted areas; (ii) to reveal the extent of the influence of fungi uptake on the substrate in a representative set of mycorrhizal mushroom *X. chrysenteron* samples, and (iii) to figure out whether site-related and temporal differences exist in the elemental composition of mushrooms of a certain species grown on contrasting substrates and collected over the extended period.

## Materials and methods

Mushroom and substrate soil samples were collected from three closely located sites on the forested plateau (approximately 120 km west of Prague, the capital of the Czech Republic) in July, August and September 2021, and in August and September 2022. The substrate soils in the three studied sites are developed on contrasting bedrock: granite, amphibolite and serpentinite. Detailed information on geomorphology and geology of the area is given in Krám et al. (2012, 2017, 2019), Štědrá et al. (2015, 2016), Dannhaus et al. (2018), Krám (2019), and Andronikov et al. (2023).

Matured species of *X. chrysenteron* which were in good conditions were collected in a forest dominated by the Norway spruce (*Picea abies*). Identification of the mushroom species was done visually based on information provided by relevant literature (Antonín et al., 2019; Knauerová et al., 2020) and by the online mushrooms atlas freely available at https://www.houbareni.cz/houby.php. Samples of *X. chrysenteron* have typically a flattened cap of 3–10 cm in diameter and of a dark-brown color. The upper outside part of the cap is often characteristically cracked. The cap’s flash is cream-colored. The sporophore is yellow to yellow-green, with large (up to 1–1.5 mm) pores. When the fruiting body is cut or pressed, the flesh becomes bluish. The cylindrical stipe is 5–10 cm long and 1–2 cm in diameter. The diameter of the stipe does not change significantly along the whole length of the stipe. The stipe is yellow at the top and becomes pink, burgundy to dark red towards the base. The stipe is finely flaky along its length. Such features of the stipe are very typical for the *X. chrysenteron* and distinguish it from other representatives of the *Xerocomellus* genus (Antonín et al., 2019; Knauerová et al., 2020; Šutara, 2008).

In addition to mushrooms, we collected mushroom-related substrate/soil samples down to the depth of 10–12 cm (the topsoil layer of 10–12 cm depth is the major source of the accumulated nutrients and elements for mycorrhizal fungi; Borovička, Konvalinková, et al., 2019) following the collecting strategy by Falandysz et al. (2017), Đurđić et al. (2021), and Andronikov et al. (2023). The studied mushrooms are ectomycorrhizal and not saprobic, therefore the surface soil layer, containing fresh litter and not playing any significant role in the accumulation of nutrients and elements, was removed. At each locality, we collected soils of two types: the soil located directly under the mushroom sample (mushroom-bearing soil likely affected by interaction with mushrooms), and the soil 1.5–2 m away from the mushroom, representing soil which was hopefully not affected by interaction with the mushroom’s mycelium (mushroom-free soil).

Techniques of sample preparation for analyses were as follows (Andronikov et al., 2023; Dryżałowska & Falandysz, 2014; Đurđić et al., 2021). Mushroom samples, after being cleaned of vegetation and soil debris, and flushed with Milli-Q water were separated into three sub-samples — the stipe, the cap, and the sporophore — weighed and then air-dried for several days. Thereafter, sub-samples of the mushroom’s fruiting bodies were dried at 65°C to constant weight. The mean percentage of mushroom’s dry matter (DM) was 9.6±4.3% in 2021 and 12.6±4.6% in 2022 for a stipe, 6.4±2.3% in 2021 and 7.8±1.6% in 2022 for a cap, and 8.9±1.8% in 2021 and 11.0±1.1% in 2022 for a sporophore. Dried fungal sub-samples were pulverized in an agate mortar. About 500 mg of each dried and pulverized sub-sample was put into the pressure-resistant and analytical quality pro-digestive polytetrafluoroethylene (PTFE) vessel. The fungal materials were pre-digested for 24 h with concentrated nitric acid (65%; Ultrapure, Romil; 7 mL) at room temperature and then further digested under pressure in a MARS 6 (CEM Corp. Matthews, NC, USA) automatic microwave digestion system. The HNO_3_-based digest, after being treated with concentrated HCl and H_2_O_2_ (both Ultrapure Romil), was diluted to 0.3N HNO_3_ using Milli-Q water for further instrumental analysis. Bulk substrate soil samples were air dried and sieved to < 2 mm fraction prior to analyses. The substrate samples have been ashed at 550°C before processing and then were homogenized and quantitatively dissolved by an HF–HClO_4_ acid digestion. The samples were then dried down and diluted in a mixture of concentrated HNO_3_ (Ultrapure Romil) and Milli-Q water in order to get 50 mL of 0.3N HNO_3_-based sample solution. All sample solutions were filtered through an acid-cleaned syringe filter with a pore size of 0.45 μm before further processing.

All sample preparation and analytical works were conducted in laboratories of the Czech Geological Survey (Prague). Elemental compositions of samples were determined with the use of the Agilent Technologies 5110 inductively coupled plasma optical emission spectrometer (ICP-OES). Samples in 0.3N HNO_3_ were introduced to plasma with a concentric glass nebulizer (Meinhard) and an Agilent Technologies SPS-4 autosampler. Operating conditions were as follows: RF power 1200 W, plasma Ar flow 12 L min^−1^, auxiliary gas flow 1.0 L min^−1^, and nebulizer gas flow 0.7 L min^−1^. Altogether, 36 individual samples (12 fruiting bodies) of mushrooms and 24 related soil samples were analyzed. The following 33 elements were measured for concentrations: Ag, Al, As, Ba, Ca, Cd, Co, Cr, Cu, Fe, Ga, K, Li, Mg, Mn, Mo, Na, Nb, Ni, P, Pb, Rb, S, Sb, Se, Sn, Sr, Ti, V, W, Y, Zn, and Zr. Sample concentrations were determined by first subtracting blank signal intensities from those obtained from the sample and standard solutions. Standard solutions consisting of known amounts of the analyzed elements were prepared using a multi-element solution (ICP 23-element standard solution IV) obtained from Merck KGaA (Germany) and mono-element standard solutions obtained from Chromservis s.r.o. (Czech Republic). The calibration was done for standard solution concentrations of 0.5; 1.0; and 2.0 mg L^−1^. A calibration curve was obtained by performing a linear least-squares regression for each element using the blank-subtracted counts and the known concentrations in each standard solution. In all cases, the regression coefficients were 0.998 or higher. For quality control, we repeatedly analyzed procedural blanks and standard reference materials (SRM) such as SRM NIST 1515 (apple leaves), SRM NIST 2709a (San Joaquin soil) and SRM NIST 2711a (Montana soil) in batches with unknown samples. The analytical error for all determinations was below 1% RSD with three replicate analyses per sample. Both procedural blanks and SRMs were processed in the same way as unknown samples. Analytical results for the standard reference materials are given in the electronic supplementary Table S1. For analyses of Ag, As, Cd, Co, S, Sb, Sn, and W which concentrations are either too low in the SRM or data were not available, we additionally used solutions of known concentrations prepared from mono-element solutions obtained from Chromservis s.r.o. (Czech Republic). Detection limits for As, P, Pb, S, Sb, Se, Sn, and W were 0.15–0.35 mg kg^−1^ DM, for Co, Ga, Mo, Ni, and Rb were 0.05–0.08 mg kg^−1^ DM, for Ag, Al, Cd, Cr, Cu, Fe, K, Mn, Na, Nb, Ti, V, Y, Zn, and Zr were 0.01–0.03 mg kg^−1^ DM, and for Ba, Ca, Li, Mg, and Sr were 0.001–0.003 mg kg^−1^ DM depending on sample weight and volume.

In order to characterize the chemical environment, we estimated elemental relations between the substrate and a mushroom’s fruiting body as well as within-mushroom elemental relations. For this, bioaccumulation (BF) and translocation (TF) factors were determined (see Busuioc et al., 2011; Dryżałowska & Falandysz, 2014; Malinowska et al., 2004).

The BF allows estimating whether a mushroom excludes or accumulates the element, it is calculated on the dry matter basis and it is expressed by the Eq.:

$$\textrm{BF}={\textrm{C}}_{\textrm{M}}/{\textrm{C}}_{\textrm{S}}$$

where C_M_ is the element’s concentration in the mushroom (in a stipe, cap, sprophore or mean fruiting body concentration), and C_S_ is the element’s content in the substrate where mushrooms take up nutrients from. The condition for a mushroom to be considered an accumulating biosystem is BF >1.

The TF allows for estimating mobility of the elements within the fruiting body. The TF is calculated on the dry matter basis according to the Eq.:

$$\textrm{TF}={\textrm{C}}_{\textrm{cap}}/{\textrm{C}}_{\textrm{stipe}}$$

where C_cap_ is the element’s concentration in the cap (or sporophore) and C_stipe_ is the element’s concentration in the stipe. The value of TF >1 would suggest high mobility of the element.

## Results

 Analyzed mushrooms and substrate samples displayed variable elemental characteristics. Some elements (Co, Ga, Mo, Sb, V, Y, and Zr) available in soils, were present in mushrooms in very low amounts, often below the detection limits (bdl) of the method while others (e.g., Se) were abundant in mushrooms, but displayed very low concentrations (down to bdl) in soil samples. The elemental composition of mushroom and soil samples collected from the three studied sites are given in Tables 1 and 2, and in the electronic supplementary Tables S2 and S3.

**Table 1:** Mean element concentrations in the substrate soil samples (mg kg^−1^)

	With mushrooms *n*=4	SD	No mushrooms *n*=4	SD
Granite-based site				
Ag	0.71	0.24	1.09	0.38
Al	33,655	4287	35,200	2890
As	11.7	1.3	11.4	1.2
Ba	83.8	10.6	91.3	16.0
Ca	1385	235	1410	208
Cd	0.35	0.06	0.52	0.08
Co	1.33	0.51	1.37	0.62
Cr	18.8	1.3	18.3	1.4
Cu	20.7	6.5	20.4	5.7
Fe	8110	430	7715	698
Ga	11.9	3.2	11.7	2.4
K	14,025	910	15,175	1012
Li	237	29	234	28
Mg	1060	101	1090	115
Mn	114	40	110	39
Mo	1.21	0.22	1.13	0.24
Na	5195	130	5275	143
Nb	10.1	2.3	9.21	2.02
Ni	7.96	1.37	8.10	1.64
P	710	56	690	84
Pb	142	17	135	27
Rb	455	28	535	36
S	910	328	910	324
Se	bdl	N/A	bdl	N/A
Sn	11.2	3.8	11.2	3.4
Sr	19.8	2.6	19.2	2.8
Ti	1630	246	1630	347
V	25.6	1.9	24.8	2.2
W	4.70	0.89	4.44	0.88
Y	4.02	1.14	3.87	0.95
Zn	50.3	6.3	46.7	7.2
Zr	23.2	3.9	23.7	6.6
Amphibolite-based site				
Ag	0.85	0.47	1.22	0.67
Al	51,185	3942	59,620	3100
As	43.8	4.0	51.0	7.1
Ba	270	15	310	45
Ca	21,070	995	21,620	1079
Cd	0.77	0.03	1.06	0.06
Co	4.40	0.69	5.27	0.48
Cr	85.9	8.5	97.4	6.8
Cu	48.5	7.6	50.5	5.5
Fe	40,280	3555	51,445	5063
Ga	17.5	0.5	19.4	0.8
K	9548	189	10,212	263
Li	57.7	10.4	63.4	7.7
Mg	22,795	1692	23,890	2001
Mn	620	27	785	53
Mo	0.55	0.19	0.60	0.23
Na	7905	733	8140	641
Nb	10.7	2.2	10.2	1.8
Ni	61.2	4.5	75.2	7.7
P	705	196	705	195
Pb	60.3	16.6	62.6	14.1
Rb	90.9	20.0	110	16
S	615	236	645	238
Sb	2.90	0.28	3.10	0.27
Se	bdl	N/A	bdl	N/A
Sn	3.40	0.43	3.57	0.65
Sr	89.8	9.5	84.7	9.4
Ti	4605	376	5095	393
V	115	5	135	8
W	1.89	0.26	2.13	0.26
Y	9.75	0.80	9.83	1.14
Zn	115	9	125	10
Zr	24.1	4.2	24.4	4.1
Serpentinite-based site				
Ag	0.27	0.10	0.34	0.13
Al	52,675	2622	58,740	3059
As	22.3	4.8	24.2	4.4
Ba	110	28	125	27
Ca	26,515	4691	26,990	5102
Cd	0.73	0.25	1.03	0.38
Co	22.3	3.0	24.5	3.9
Cr	155	19	165	15
Cu	33.3	23.4	35.3	27.5
Fe	42,575	2411	50,690	3081
Ga	14.3	0.9	15.6	0.9
K	5945	2931	6470	3108
Li	44.6	15.2	50.7	17.8
Mg	41,500	4434	43,195	4825
Mn	430	85	505	98
Mo	bdl	N/A	bdl	N/A
Na	6755	1563	6950	1602
Nb	6.06	1.52	5.93	1.24
Ni	230	17	250	22
P	445	179	445	205
Pb	48.6	5.9	48.4	9.5
Rb	60.5	30.6	73.1	37.0
S	1275	53	1195	35
Sb	1.88	0.46	1.97	0.38
Se	bdl	N/A	bdl	N/A
Sn	2.69	0.42	2.72	0.45
Sr	76.6	14.0	80.2	12.5
Ti	2515	320	2590	290
V	125	18	130	18
W	1.92	0.56	2.05	0.61
Y	10.1	1.6	10.1	1.1
Zn	105	39	115	44
Zr	14.2	2.9	15.2	2.8

*bdl*, below the detection limit; *N/A*, not applicable

n, a total number of soil samples analyzed

**Table 2:** Mean element concentrations in the analyzed *Xerocomellus chrysenteron* samples (mg kg^−1^ DM)

				GRANITE-BASED SITE				
*FB part	Stipe	SD	Cap	SD	Spor	SD	Bulk FB	SD
(%)	(40.7)	(13.2)	(29.1)	(4.8)	(30.2)	(16.5)	*n*=4	
Elements								
Ag	1.04	0.38	1.75	0.38	4.09	2.39	2.36	1.03
Al	5.15	3.10	6.37	4.44	13.3	8.6	7.37	3.03
As	0.67	0.25	1.33	0.81	2.18	1.60	1.37	0.81
Ba	0.55	0.31	0.33	0.09	0.31	0.15	0.40	0.19
Ca	94.3	25.5	65.0	6.8	145	47	105	29
Cd	2.09	1.10	3.20	1.68	7.69	4.14	4.12	2.34
Co	0.11	0.01	0.12	0.01	0.13	0.02	0.11	0.01
Cr	0.09	0.05	0.12	0.08	0.24	0.21	0.13	0.08
Cu	15.1	5.7	23.9	13.9	44.9	15.3	26.2	6.4
Fe	20.4	10.5	29.7	10.7	62.0	40.6	34.7	18.5
Ga	bdl	N/A	bdl	N/A	bdl	N/A	bdl	N/A
K	22,855	5505	32,430	5990	17,950	5029	24,050	4410
Li	0.22	0.24	0.18	0.20	0.05	0.06	0.17	0.19
Mg	415	72	620	105	1015	209	675	82
Mn	5.72	1.35	6.51	0.69	9.57	0.66	7.24	0.28
Mo	0.14	0.03	0.17	0.05	0.22	0.09	0.17	0.05
Na	330	230	570	460	310	252	390	299
Nb	0.19	0.13	0.20	0.10	0.13	0.06	0.18	0.10
Ni	0.42	0.08	0.55	0.13	0.70	0.24	0.54	0.12
P	2935	963	4305	1576	7865	1913	4895	1093
Pb	0.47	0.26	0.35	0.04	0.41	0.15	0.40	0.14
Rb	1620	284	2595	359	2120	638	2075	407
S	2980	1824	4170	2220	6345	3904	4480	2944
Sb	bdl	N/A	bdl	N/A	bdl	N/A	bdl	N/A
Se	0.61	0.12	0.91	0.35	1.48	0.45	0.92	0.16
Sn	0.44	0.16	0.51	0.20	0.49	0.15	0.48	0.14
Sr	0.34	0.17	0.21	0.07	0.50	0.30	0.37	0.18
Ti	0.17	0.28	0.27	0.46	0.55	1.02	0.23	0.39
V	0.05	0.01	0.07	0.01	0.09	0.03	0.07	0.00
W	0.59	0.36	0.93	0.46	1.20	0.39	0.88	0.33
Y	bdl	N/A	bdl	N/A	bdl	N/A	bdl	N/A
Zn	72.5	29.3	105	32	140	38	105	28
Zr	bdl	N/A	bdl	N/A	bdl	N/A	bdl	N/A
				Amphibolite-based site				
*FB part	Stipe	SD	Cap	SD	Spor	SD	Bulk FB	SD
(%)	(29.7)	(8.6)	(37.0)	(16.4)	(33.3)	(12.6)	*n*=4	
Elements								
Ag	1.37	0.80	2.03	1.06	5.33	1.49	3.06	1.12
Al	9.26	1.80	24.2	24.3	18.6	15.6	19.5	17.7
As	0.90	0.33	1.34	0.96	1.80	0.80	1.33	0.62
Ba	0.89	0.81	0.67	0.42	0.70	0.47	0.75	0.59
Ca	93.3	40.3	127	66	87.2	49.1	103	52
Cd	2.59	1.70	3.88	2.90	9.53	4.39	5.43	2.57
Co	0.13	0.02	0.11	0.01	0.14	0.03	0.12	0.01
Cr	0.27	0.30	0.26	0.18	0.33	0.23	0.30	0.24
Cu	22.8	21.9	26.8	21.8	51.0	16.3	35.2	19.0
Fe	12.1	3.8	13.5	5.9	24.6	10.4	17.7	6.7
Ga	bdl	N/A	bdl	N/A	bdl	N/A	bdl	N/A
K	20,175	1605	32,090	7403	16,940	2858	24,030	6044
Li	0.16	0.08	0.14	0.05	0.10	0.09	0.13	0.07
Mg	480	204	605	132	1035	61	720	140
Mn	11.4	7.2	10.7	6.1	11.8	5.2	11.7	5.6
Mo	0.12	0.03	0.15	0.05	0.18	0.09	0.15	0.06
Na	570	236	425	140	270	115	435	142
Nb	0.23	0.11	0.27	0.06	0.29	0.11	0.26	0.08
Ni	0.52	0.42	0.56	0.40	0.61	0.42	0.58	0.40
P	3310	1701	3920	585	7675	985	5120	1001
Pb	0.56	0.46	0.58	0.48	0.37	0.09	0.52	0.36
Rb	415	98	755	290	600	215	615	206
S	3370	1902	4095	2554	5915	4521	4675	3361
Sb	bdl	N/A	bdl	N/A	bdl	N/A	bdl	N/A
Se	1.92	1.29	1.48	1.10	1.54	0.92	1.58	0.97
Sn	0.42	0.12	0.46	0.09	0.56	0.33	0.50	0.16
Sr	0.69	0.74	0.57	0.37	0.45	0.41	0.59	0.53
Ti	1.80	3.37	4.59	8.85	6.53	12.76	3.99	7.67
V	0.22	0.25	0.14	0.11	0.21	0.21	0.19	0.19
W	0.68	0.53	0.59	0.23	1.41	0.51	0.93	0.30
Y	bdl	N/A	bdl	N/A	bdl	N/A	bdl	N/A
Zn	84.5	64.0	95.8	44.3	168	47	120	38
Zr	bdl	N/A	bdl	N/A	bdl	N/A	bdl	N/A
				Serpentinite-based site				
*FB part	Stipe	SD	Cap	SD	Spor	SD	Bulk FB	SD
(%)	(37.4)	(6.8)	(27.6)	(9.5)	(35.1)	(10.2)	*n*=4	
Elements								
Ag	0.31	0.17	0.68	0.33	2.29	1.46	1.06	0.57
Al	5.68	2.30	14.7	5.7	9.45	3.56	9.27	2.52
As	0.51	0.01	0.65	0.20	1.02	0.25	0.72	0.11
Ba	0.39	0.31	0.85	0.48	0.44	0.32	0.54	0.30
Ca	85.4	33.3	140	30	114	38	110	20
Cd	0.26	0.13	0.52	0.30	1.60	0.78	0.77	0.33
Co	0.35	0.32	0.33	0.29	0.34	0.33	0.33	0.31
Cr	0.21	0.10	0.25	0.14	0.25	0.06	0.23	0.10
Cu	8.83	1.26	13.2	4.55	33.4	7.8	18.3	4.2
Fe	20.5	20.7	28.7	21.9	35.8	23.3	28.3	22.8
Ga	bdl	N/A	bdl	N/A	bdl	N/A	bdl	N/A
K	17,925	8209	24,825	14,801	14,520	5491	18,495	8001
Li	0.53	0.55	0.46	0.35	0.56	0.49	0.50	0.39
Mg	375	21	545	207	905	224	600	117
Mn	8.59	4.63	9.63	7.30	7.30	2.75	8.05	3.02
Mo	0.10	0.02	0.12	0.02	0.20	0.07	0.14	0.04
Na	220	126	245	214	240	118	235	143
Nb	0.19	0.09	0.22	0.11	0.20	0.10	0.20	0.09
Ni	0.69	0.57	0.83	0.43	0.91	0.41	0.78	0.39
P	1720	352	2460	984	5900	1982	3300	827
Pb	0.48	0.25	0.37	0.10	0.32	0.02	0.40	0.12
Rb	380	150	640	317	510	144	495	166
S	3655	2685	5330	3977	6695	3657	5160	3602
Sb	bdl	N/A	bdl	N/A	bdl	N/A	bdl	N/A
Se	1.65	1.61	3.33	1.70	5.92	4.11	3.52	1.65
Sn	0.38	0.06	0.42	0.13	0.39	0.11	0.40	0.07
Sr	0.56	0.45	1.04	0.72	0.57	0.30	0.67	0.41
Ti	1.09	1.53	1.47	1.66	1.38	1.56	1.28	1.58
V	0.08	0.02	0.11	0.05	0.11	0.02	0.10	0.03
W	0.86	0.72	0.72	0.21	1.01	0.35	0.85	0.35
Y	bdl	N/A	bdl	N/A	bdl	N/A	bdl	N/A
Zn	99.1	75.2	86.5	30.9	121	40	100	35
Zr	bdl	N/A	bdl	N/A	bdl	N/A	bdl	N/A

*Information on the percentage (with SD) of the certain fruiting body (FB) part

*Bulk FB* bulk fruiting body, *bdl* below the detection limit, *N/A* not applicable, *Spor* sporophore

n, a total number of the fruiting bodies (each consisting of three subsamples) analyzed

Elements such as Ca, Mg, Na, P, S, and Zn are essential for the living organism’s growth (Kaspari & Powers, 2016; Zocher et al., 2018), and their concentrations in the mushrooms studied were high (from hundreds to thousands mg kg^−1^ DM). Other elements with concentrations ranging from hundreds to thousands mg kg^−1^ DM can be both essential and non-essential. Due to the toxicity or potential toxicity of some elements (e.g., elements such as Cd, Cr, and Se), their elevated concentrations in mushrooms might pose a health risk to humans.

### Soils

Soil samples developed on contrasting bedrock displayed different elemental compositions (Tables 1 and S2). For both mushroom-free and mushroom-bearing soil types, amphibolite- and serpentinite-based soils were characterized by high concentrations of major elements such as Al, Ca, Fe, and Na, and trace elements such as Cd, Mn, Sr, V, and Zn. Granite-based soils displayed much lower concentrations of both major and trace elements. On the other hand, the granite-based soils were characterized by higher concentrations of K, Li, Rb, and Pb compared to both the amphibolite- and serpentinite-based soils. The amphibolite-based soils displayed higher concentrations of Ti, As, and Ba than both granite- and serpentinite-based soils. The serpentinite-based soils were richer in Mg, Co, Cr, and Ni than both granite- and amphibolite-based soils.

Concentrations of some elements in soils displayed measurable temporal variations. Among those, As, Fe, Mn and Nb are with higher concentrations in granite-based soil samples collected in July 2021 (up to 1.5–2 times) than in soils collected from the same site during the rest of the period of observation. Notable differences can be seen for Cu, whose concentrations were 1.5–2 times lower in granite- and amphibolite-based soil samples, and are up to three times higher in serpentinite-based soil samples all collected in July 2021 than in samples collected from the same locations during the rest of the period of observation. Lead displays 2–2.5 times lower concentrations in amphibolite-based soil samples collected in July 2021 than in samples collected during the rest of the period of observation. Concentrations of Sb in granite-based soils collected in August 2021 are 1.5 times higher than those collected during the rest of the period of observation. Concentrations of Sn and W in granite-based soils collected in September 2022 are 1.5–2 times lower than in those collected during the rest of the period of observation. For serpentinite-based soils, concentrations of Ca, Cd, Li, Na, Sr, and Zn are 1.5–2.5 times higher for samples collected in July 2021 than for samples collected during the rest of the period of observation.

### Mushrooms

All studied mushroom samples contained different elements in amounts which are mostly within the frames reported by, e.g., Kalač and Svoboda (2000), Svoboda et al. (2006), Svoboda and Chrastný (2008), Kalač (2009, 2010), Mallikarjuna et al. (2013), Kojta et al. (2016), Zocher et al. (2018), Borovička, Braeuer, et al. (2019), Andronikov et al. (2022, 2023) for different mushroom’s genera and species. Overall, bulk mushrooms (bulk compositions of the mushrooms fruiting bodies for individual samples were calculated according to the weight proportions of individual mushrooms parts) were characterized by variable concentrations of major, minor, and trace essential and non-essential elements (Table 2 and electronic supplementary Table S3).

As well as in the case of the substrate soils, some temporal tendencies were observed in the elemental composition of the studied mushrooms. Mushroom samples collected in July 2021 often displayed higher concentrations of most elements compared to samples collected during the rest of the study period (like in substrate soils). Additionally, mushroom samples collected in September 2021 displayed high concentrations of some elements (unlike in the case of the substrate soils). It is not clear yet why samples collected during these two sampling sessions were compositionally different from other samples.

Although most elements in mushrooms did not show pronounced site-dependency, some of them (Rb, Ag, As, and Se) displayed marked lateral differences in concentrations. Rubidium was more abundant in the mushrooms from the granite-based site (1600–2500 mg kg^−1^) than from the two other sites (271–873 mg kg^−1^), which is consistent with much higher concentrations of Rb in the granite-based soils (418–577 mg kg^−1^) than in soils from the two other sites (47–128 mg kg^−1^). Concentrations of Ag were similar in mushrooms from granite- and amphibolite-based sites (1.53–4.55 mg kg^−1^), but they were lower in samples from the serpentinite-based site (0.36–2.34 mg kg^−1^). It is consistent with higher concentrations of Ag in soils from the granite- and amphibolite-based sites (0.55–2.57 mg kg^−1^) than in soils from the serpentinite-based site (0.14–0.45 mg kg^−1^). A similar tendency was observed for As (up to 4.4 mg kg^−1^ in samples from the granite- and amphibolite-based sites, and only 0.58–1.15 mg kg^−1^ in mushrooms from the serpentinite-based site). It is not however completely consistent with concentrations of As in soils because the lowest As concentrations were measured in soils from the granite-based site (10–13 mg kg^−1^) and the highest in soils from the amphibolite-based site (38–53 mg kg^−1^). Arsenic did not show signs of hyperaccumulation (process of uptake, translocation and accumulation of elements at concentrations significantly higher than in

surroundings; Maestri et al., 2010) in the *X. chrysenteron* samples presently studied that is consistent with observations made by Walenta et al. (2023) that no As hyperaccumulation occurred in commonly eaten mushrooms of Central Europe. Selenium concentrations were much higher in mushroom samples collected from the serpentinite-based site (up to 5.6 mg kg^−1^) than from the granite- (up to 1.1 mg kg^−1^) and amphibolite-based (up to 2.6 mg kg^−1^) sites, although concentrations of the element were similarly low in soils from all three sites (bdl).

## Discussion

**Fig. 1:**
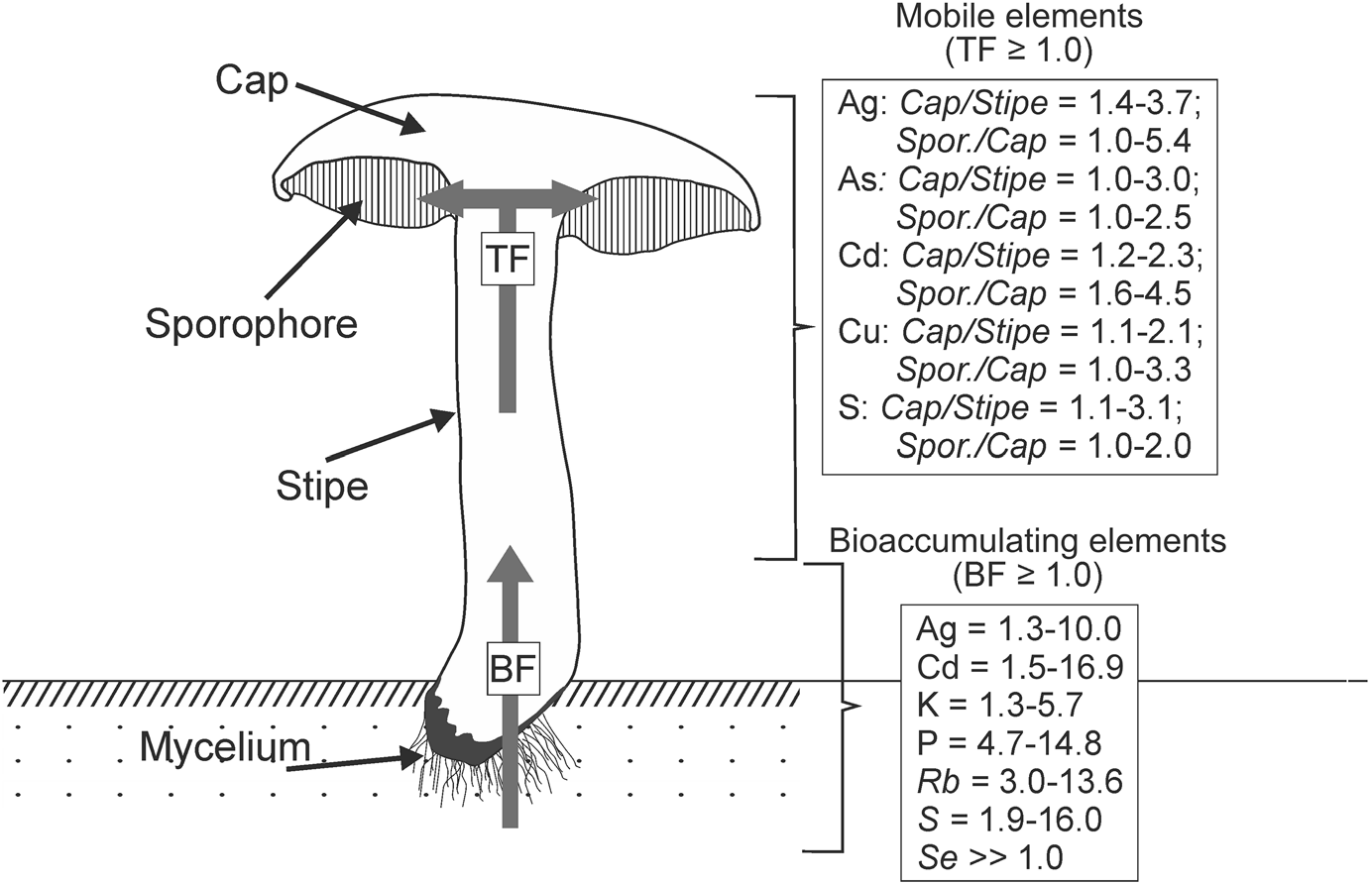
A sketch showing tendencies in elements bioaccumulation (BF, bioaccumulation factor) from the substrate and in within-mushroom elements translocation (TF, translocation factor). Bioaccumulating elements have BF ≥ 1.0. Mobile elements have TF ≥ 1.0. Spor., sporophore. See text for more details

Ability of mushrooms to accumulate or exclude elements can be estimated with the use of the bioaccumulation factor (BF). Our results showed that Ag, K, P, Rb, and S were always enriched (accumulated) in the mushroom’s fruiting bodies (BF varied from 1.1 for K in the mushroom sample from the serpentinite-based site collected in August 2022 to 11.9 for P in the mushroom sample collected from the same site and at the same time) (Fig. 1; Table S4). Cadmium which belongs among the most frequently determined elements in mushrooms due to its deleterious effects on human health (Borovička et al., 2022) was enriched in mushrooms collected from all three sites (BF = 1.5–16.9) except for samples collected in July and August 2021 from the serpentinite-based site (BF = 0.44–0.55). Mushrooms studied can behave both as bioaccumulating and as bioexcluding systems with respect to Cu and Zn (BF varied between 0.3 and 2.8 over the course of the observation). Most other elements were bioexluded or near bioexcluded showing the BF values below unity regardless of the site or the season (Table S4). Significant variations in interannual bioaccumulation activities of the Boletaceae mushrooms were noted by Zhang et al. (2010) and Chojnacka et al. (2013). Such variations in mushrooms grown on a substrate of a stable composition (Andronikov et al., 2020; Hruška & Krám, 2003; Kopáček et al., 2016; Krám et al., 2019) could be due to biological factors and/or fluctuations in seasonal and annual weather conditions. These factors should be taken into consideration during further works with mushroom samples. Interestingly, Se in all analyzed mushroom samples was significantly enriched relative to the substrate (up to 5.6 mg kg^−1^ in a sample from the serpentinite-based site collected in September 2021; Table 2 and S3), but since concentrations of Se in substrate soils were below the detection limit in all samples (Tables 1 and S2), no BF_Se_ has been calculated. High concentrations of Se in fungi relative to the substrate are not a new phenomenon and have already been described for both edible (especially *Boletus* spp.) and non-edible (especially *Amanita* spp.) mushrooms (Cocchi et al., 2006; Falandysz, 2008; Jorhem & Sundström, 1995; Komárek et al., 2007). We can suggest on the example of Se that the mushrooms studied took up only limited and necessary for a life circle amounts of elements almost regardless of the substrate composition. The fact that most elements in mushrooms did not show significantly pronounced site-dependency, could be in favor of such a suggestion (see e.g., Borovička, Konvalinková, et al., 2019; Tlalka et al., 2008; Walker & White, 2018).

Within-mushroom distribution of elements (translocation) indicates the element’s mobility. Preferable accumulation of any element in the stipe would point to the element’s low mobility whereas translocation to and accumulation in apical parts of the fruiting body would suggest high mobility of the element. Only several elements analyzed have displayed constant high mobility and preferable accumulation in the mushroom’s apical parts (Fig. 1; Table S5). In the case of almost all highly mobile elements, sporophore was the main accumulator of the elements. Elements such as Ag, As, Cd, Cu, and S showed such a tendency regardless of the season and sample location (TF_cap/stipe_ = 1.0–3.7 and TF_spor/cap_ = 1.0–5.4). Other elements could show site-dependency in terms of the translocation. Zinc, Fe, Mg, and P behaved the same way as Ag, As, Cd, Cu, and S except for Zn in samples from the amphibolite- and serpentinite-based sites collected in July 2021 (TF_cap/stipe_ = 0.4–0.9 and TF_spor/cap_ = 1.1–1.5), for Fe and P in samples from the amphibolite-based site collected at the same time (TF_cap/stipe_ = 0.5–0.8 and TF_spor/cap_ = 1.5–2.3), and for Mg in samples from the serpentinite-based site also collected in July 2021 (TF_cap/stipe_ = 0.8 and TF_spor/cap_ = 2.4). Elements such as K and Rb, mobile though, preferably accumulated in the cap, but not in the sporophore (TF_cap/stipe_ = 1.1–2.6 and TF_spor/cap_ = 0.5–1.0). Such behavior of K and Rb was observed for all samples except for those collected from the serpentinite-based site in July 2021 (TF_cap/stipe_ = 0.6–0.7; TF_spor/cap_ = 1.0–1.4). Calcium and Sr behaved differently for samples collected from different sites. In the case of the granite-based site, the elements preferably accumulated in the stipe and sporophore, but not in the cap (TF_cap/stipe_ = 0.4–0.9; TF_spor/cap_ = 1.3–3.3). For samples collected from the amphibolite- and serpentinite-based sites, the elements preferably accumulated in the cap, but not in the stipe and sporophore (TF_cap/stipe_ = 1.1–5.7; TF_spor/cap_ = 0.5–1.0). Sodium also displayed site-dependent within-mushroom distribution. For the granite-based site, Na preferably accumulated in the cap (TF_cap/stipe_ = 1.4–2.1 and TF_spor/cap_ = 0.5–0.7) whereas Na showed low mobility accumulating in the stipe for the amphibolite-based site (TF_cap/stipe_ = 0.4–0.9 and TF_spor/cap_ = 0.3–0.9). Sodium behaved erratically for samples collected from the serpentinite-based site. Elements such as Co, Cr, Li, Mo, Nb, Pb, Sn, and V showed different within-mushroom distribution styles, but because of very low concentrations of these elements, any within-mushroom features cannot be estimated reliably. The rest of the analyzed elements (Al, Ba, Mn, Ni, Se, Ti, and W) behaved erratically not showing any pronounced tendencies in within-mushroom distributions. As well as in the case of the BF, we can suggest that variations in TF could be due to biological factors and/or fluctuations in seasonal and annual weather conditions. However, the findings require more thorough and detailed study.

### Mushroom-to-soil interaction

We analyzed mushroom-bearing and mushroom-free soil samples from all three sites to see whether and how mushrooms uptake influenced (if at all) the elemental composition of the substrate. Since the studied area was not affected significantly by modern industrial pollution, the elemental composition of the analyzed soil horizons was supposed to remain mostly unchanged throughout the period of the observation (a total duration of fifteen months) (Andronikov et al., 2020; Hruška & Krám, 2003; Kopáček et al., 2016; Krám et al., 2019; Zhang et al., 2010). However, we identified some features in elemental relation between the mushroom-bearing and mushroom-free soils. Although most elements behaved erratically with respect to the soil type, Ag, Cd, Rb, Al, and K displayed strong systematic depletion in the mushroom-bearing soils relative to the mushroom-free soils (significant correlation with the Pearson’s correlation coefficient *R* = 0.971 to 0.999 and *p* < 0.01). Taking into account low concentrations of Ag and Cd in the mushroom-free soils (0.14–2.12 mg kg^−1^ Ag and 0.28–1.40 mg kg^−1^ Cd) and their relatively high concentrations in bulk mushroom’s fruiting bodies (0.36–4.55 mg kg^−1^ Ag and 0.40–8.33 mg kg^−1^ Cd), it seems plausible to suggest that the mushroom’s uptake can influence concentrations of the elements in the substrate soils. Concentrations of Rb in the mushroom-free soils were not very high (47–580 mg kg^−1^ Rb), and uptake of significant amounts of Rb (concentrations of Rb in mushrooms bulk fruiting bodies varied between 270 and 2490 mg kg^−1^) might lead to the depletion of soils in the element. Aluminum was always depleted in the mushroom-bearing soils compared to the mushroom-free soils, but concentrations of the element in the mushroom’s fruiting bodies (4.9–45.4 mg kg^−1^) were almost four orders of magnitude lower than Al concentrations in the soils (3.5–6.3 wt%). It is very unlikely therefore that the uptake of insignificant amounts of the element could directly influence its concentration in the element-rich substrate. However, a systematic character of Al depletion in the mushroom-bearing soils could point to the involvement of additional factors in the change of the mushroom-bearing soils’ compositions. Similar to Al, potassium is a major element in soils studied (0.4–1.6 wt% K), but, unlike Al, potassium was also a major element in the mushroom’s fruiting bodies (1.1–3.3 wt% K). Since mushrooms took up high amounts of K necessary for their existence, uptake could lead to depletion of the affected substrate in the element.

Arsenic showed not systematic and not strong though, but notable depletion in the mushroom-bearing soils from the amphibolite- and serpentinite-based sites. Although As was depleted in mushroom-bearing soils from the amphibolite- and serpentinite-based sites (significant correlation with *R* = 0.976 and *p* < 0.01), such depletion has been mostly numerically insignificant (2–8%). Only in September 2021 and 2022, As in samples of the mushroom-bearing soils from the amphibolite-based site showed high degrees of depletion (15–25%). In August and September 2021, As showed high degrees of depletion (13–15%) for samples from the serpentinite-based site. Since the concentration of As in mushrooms was only about 5–10% of concentrations in the soils, it does not seem that uptake alone would change the concentration of the element in the substrate significantly.

Iron and Mn were depleted in mushroom-bearing soils collected from the amphibolite- and serpentinite-based sites (significant correlation with *R* = 0.865–0.991 and *p* < 0.01), and enriched in samples from the granite-based site. Since concentrations of both elements in the mushroom-free soils (0.7–5.8 wt% Fe and 81–825 mg kg^−1^ Mn) were much higher than those in bulk mushrooms (11–62 mg kg^−1^ Fe and 5–18 mg kg^−1^ Mn), it would be necessary to suggest involvement of additional factors to explain the change of the substrate soil Fe and Mn concentrations. Mushrooms are known to excrete weak acids to substrate (Arnott, 1995; Dusengemungu et al., 2021; Fomina et al., 2004; Gadd, 1999, 2007; Gadd et al., 2014; Landweert et al., 2001) that can change pH of the affected soil. Since both, Fe and Mn exist in the environment in several oxidation states, a change in the soil’s pH could change the oxidation state and mobility of the elements. Depletion of mushroom-bearing soils in Fe and Mn occurred for the amphibolite-based and serpentinite-based sites, but not for the granite-based site. Both amphibolite- and serpentinite-based soils are much richer in Fe and Mn than the granite-based soils (Tables 1and S2). Iron- and Mn-rich soils often contain ferromanganese nodules which are products of pedogenesis (Huang, 2022). Manganese(III)/Mn(IV) oxides in such nodules can oxidize Fe(II) forming Fe(III) precipitates (Liu et al., 2021). Variations in Fe and Mn oxidation states could significantly change the solubility and mobility of the elements and result in the depletion of the mushroom-bearing soils in redox-dependent elements. However, a detailed study of the oxidation states of the soils was beyond the scope of the present work, although it should be a subject of future studies.

Chromium, V, and W were systematically enriched in the mushroom-bearing soils from the granite-based site, but they were depleted in samples collected from the amphibolite- and serpentinite-based sites (significant correlations with *R* = 0.997, 0.734, and 0.987, respectively, and *p* < 0.01 for Cr and W, and < 0.05 for V). However, since concentrations of these elements in mushroom’s fruiting bodies were very low (Tables 1 and S2), it would be too soon to make any conclusions about the influence of mushroom’s uptake on the elements’ concentrations in substrate soils. Other minor and trace elements could have displayed both enrichment and depletion (non-systematic) in the mushroom-free soil compared to the mushroom-bearing soil, but deviations did not exceed 10% in most cases.

**Fig. 2:**
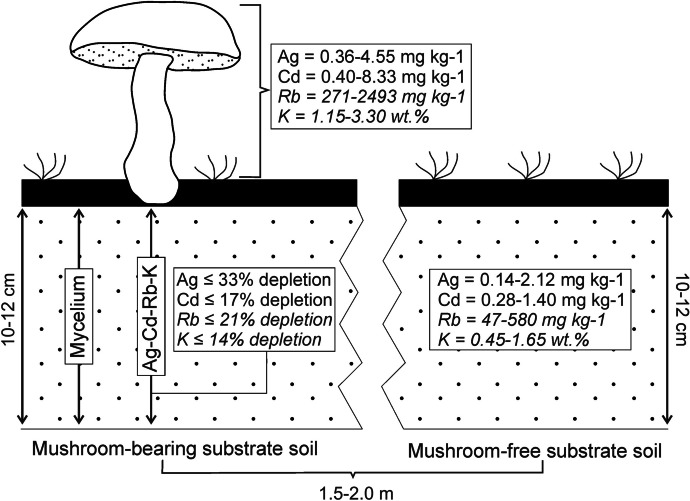
A sketch showing the possible influence of mushroom’s uptake on the composition of the substrate soil. See text for more details

In order to estimate the possible influence of mushroom’s uptake on the substrate, we chose Ag, Cd, K, and Rb, whose elements were systematically depleted in the mushroom-bearing soils (Fig. 2). We excluded Al from the consideration because its concentrations in soils were incomparably higher than in the mushrooms. A *U* test of Mann-Whitney was used to test for possible statistically significant differences between the elements’ variables. For Ag, K, and Rb, the differences were statistically not significant (*p* < 0.05) whereas for Cd, the difference was statistically significant (*p* < 0.05). Nevertheless, since depletion in the named elements of the mushroom-bearing relative to the mushroom-free soils was systematic (significant correlation with *R* = 0.971 to 0.999 and *p* < 0.01), we conducted mass-balance calculations for all four elements.

Since the depth from which mushroom mycelium takes up elements and nutrients is mostly restricted to 10–12 cm (Borovička, Konvalinková, et al., 2019), we assumed that the volume of soil affected by the uptake can be approximated as a flat cylinder with both height and diameter of 11 cm (other shapes could be assumed but they do not make much difference). We also assumed that the average specific gravity of soils would be 2.70 g/cm^3^ (typically specific gravity of soils is in the range of 2.60–2.80 g/cm^3^; Dhir et al., 2018). This way, we came to the weight of soil affected by an average *X. chrysenteron* mushroom to be 2.77 kg. From this, we calculated the amount of each considered element in the same volumes of both mushroom-free and mushroom-bearing soils (Table 3). The differences in element amounts were 0.85 mg for Ag, 0.35 mg for Cd, 130 mg for Rb, and 4040 mg for K (depletion of 14–33% for the named elements). We also calculated that an average mushroom’s fruiting body from the studied localities contained 0.02 mg Ag, 0.04 mg Cd, 10 mg Rb, and 220 mg K. Since mushrooms take elements and nutrients directly from the substrate, and taking into account the above figures, uptake by the average individual mushroom’s fruiting body would be directly responsible for only 2.5–11.5% of the total depletion of the affected substrate in the named elements. However, the mushroom’s influence on the substrate is not limited by the uptake only. Mushrooms may release organic acids to the substrate whose acids aggressively attack mineral surfaces and form metal-organic complexes (Arnott, 1995; Landweert et al., 2001; Gadd, 2007; 2017; Gadd et al., 2014; Dusengemungu et al., 2021). Most metal-organic complexes formed this way are water-soluble and could be easily washed away by groundwater or taken up by vascular plants significantly adding therefore to the depletion of the substrate in metals. This could, in particular, explain the depletion of mushroom-bearing soils in major elements whose concentrations in mushrooms are incomparably lower than in substrate. Therefore, mushrooms can deplete substrate in various elements both directly through uptake and indirectly through the formation of water-soluble metal-organic complexes.

**Table 3:** Results of the mass-balance calculations for the system soil-mushroom

	Concentration in mushroom-free soil (mg kg^−1^)	Concentration in mushroom-bearing soil (mg kg^−1^)	Amount in mushroom-free soil (mg)	Amount in mushroom-bearing soil (mg)
Ag	0.92	0.61	2.55	1.70
Cd	0.73	0.60	2.01	1.67
Rb	225	175	620	485
K	10,590	9135	29,335	25,300
	Differences between two soil types (mg)	Concentration in mushroom fruiting body (mg kg^−1^)	Amount in mushroom fruiting body (mg)	
Ag	0.85	2.19	0.022	
Cd	0.34	3.90	0.039	
Rb	130	1025	10.2	
K	4040	22,245	220	

Mean calculated weight of a mushroom’s fruiting body is 10 g (DM);

Mean weight of the soil volume affected by mushroom’s uptake is estimated as 2.77 kg;

See text for more details

It follows from the data obtained that mushrooms can directly influence concentrations of Ag, Cd, K, and Rb in the substrate soils via uptake, and indirectly via release of weak organic acids to the substrate (additionally, elements such as Al, As, Fe, Mn, and probably Co, Cr, Ni, V, and W). However, *X. chrysenteron* happened not to be such an efficient accumulator of heavy metals (including toxic) as *Boletus* spp. (Andronikov et al., 2023; Cocchi et al., 2006; Jorhem & Sundström, 1995; Komárek et al., 2007), let alone non-edible mushrooms of *Amanita*, *Galerina*, *Sarcosphaera*, and *Thelephora* genera (Borovička et al., 2022; Braeuer et al., 2020; Damodaran et al., 2014; Falandysz, 2008). It is obvious that it is necessary to conduct a more detailed and extended study of the mushroom’s uptake influence on the composition of the substrate.

## Conclusions

The current study showed that fruiting bodies of the *X. chrysenteron* were enriched in Ag, Cd, K, P, Rb, S, Se, and Zn relative to the substrate. Specific geochemistry of the bedrock was almost not reflected in the compositions of the mushroom’s fruiting bodies with the exception of Ag, As, Rb, and Se which showed site-dependency to a larger or lesser extent. Site-independency for most analyzed elements suggests that mushrooms took up only limited and necessary for a life circle amounts of elements almost regardless of the substrate composition.

Temporal features observed in the elemental composition of the mushrooms were mostly not systematic and varied depending on each element. Only for samples collected in July 2021 (both mushrooms and soils) and to a lesser extent in September 2021 (mushrooms only), a significant number of elements displayed higher concentrations compared to samples collected during the rest of the period of observation.

Only Ag, As, Cd, Cu, and S have systematically and Fe, Mg, P, Rb, and Zn often displayed high within-mushroom mobility with the tendency to preferably accumulate in the apical mushroom parts regardless of the season and the mushroom’s location. The rest of the elements mostly behaved erratically not showing pronounced within-mushroom tendencies.

Mushroom-bearing soils were systematically depleted in Ag, Cd, K, and Rb, relative to the mushroom-free soils (14–33%). Several other elements also displayed depletion in mushroom-bearing soils, but not systematically. Uptake by an average mushroom could be directly responsible for only 2.5–11.5% of a total depletion of the affected substrate in each of the named elements.

### Supplementary information

ESM 1 (XLS 55 kb)

ESM 2 (XLS 48 kb)

ESM 3 (XLS 60 kb)

ESM 4 (XLS 60 kb)

ESM 5 (XLS 46 kb)

## Data Availability

The datasets used and analyzed during the current study are available from the corresponding author on reasonable request.
